# Dirammox-dominated microbial community for biological nitrogen removal from wastewater

**DOI:** 10.1007/s00253-024-13214-2

**Published:** 2024-06-21

**Authors:** Yu Hu, Yulin Wang, Runhua Wang, Xiaokang Wang, Shuang-Jiang Liu

**Affiliations:** 1 State Key Laboratory of Microbial Technology, Shandong University, Qingdao, P. R. China; 2 State Key Laboratory of Microbial Resources, Institute of Microbiology, Chinese Academy of Sciences, Beijing, P. R. China; 3 University of Chinese Academy of Sciences, Beijing, P. R. China

**Keywords:** Dirammox, Activated sludge, *Alcaligenes*, Ammonia removal

## Abstract

**Abstract:**

Direct ammonia oxidation (Dirammox) might be of great significance to advance the innovation of biological nitrogen removal process in wastewater treatment systems. However, it remains unknown whether Dirammox bacteria can be selectively enriched in activated sludge. In this study, a lab-scale bioreactor was established and operated for 2 months to treat synthetic wastewater with hydroxylamine as a selection pressure. Three Dirammox strains (*Alcaligenes aquatilis* SDU_AA1, *Alcaligenes aquatilis* SDU_AA2, and *Alcaligenes* sp. SDU_A2) were isolated from the activated sludge, and their capability to perform Dirammox process was confirmed. Although these three Dirammox bacteria were undetectable in the seed sludge (0%), their relative abundances rapidly increased after a month of operation, reaching 12.65%, 0.69%, and 0.69% for SDU_A2, SDU_AA1, and SDU_AA2, respectively. Among them, the most dominant Dirammox (SDU_A2) exhibited higher nitrogen removal rate (32.35%) than the other two strains (13.57% of SDU_AA1 and 14.52% of SDU_AA2). Comparative genomic analysis demonstrated that the most dominant Dirammox bacterium (SDU_A2) possesses fewer complete metabolic modules compared to the other two less abundant *Alcaligenes* strains. Our findings expanded the understanding of the application of Dirammox bacteria as key functional microorganisms in a novel biological nitrogen and carbon removal process if they could be well stabilized.

**Key points:**

*• Dirammox-dominated microbial community was enriched in activated sludge bioreactor.*

*• The addition of hydroxylamine played a role in Dirammox enrichment.*

*• Three Dirammox bacterial strains, including one novel species, were isolated.*

**Graphical abstract:**

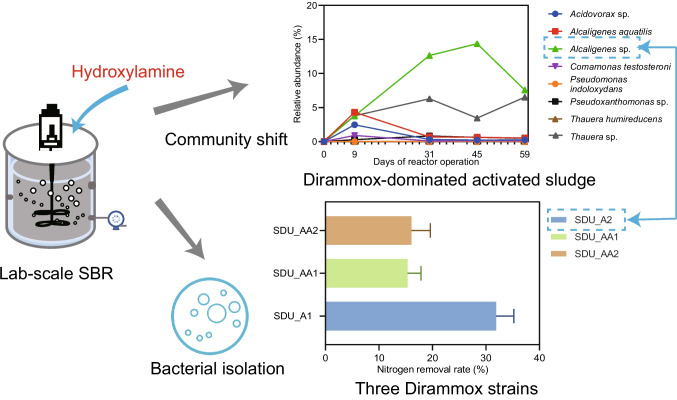

**Supplementary Information:**

The online version contains supplementary material available at 10.1007/s00253-024-13214-2.

## Introduction

Nitrogen removal is one of the critical purposes for the design of wastewater treatment systems. The conversion between dinitrogen gas and bioavailable nitrogen (“reactive”) on our planet is predominantly governed by microorganisms (Kuypers et al. 2018; Stein and Klotz 2016). Advancements in understanding the physiological and ecological characteristics of nitrogen-cycling microorganisms have contributed to the emergence of novel biological treatment processes. Sequential nitrification and denitrification, which form the basis of conventional nitrogen removal systems, have been widely adopted in wastewater treatment plants (WWTPs) (Rahimi et al. 2020; Thakur and Medhi 2019). The discovery and characterization of anaerobic ammonium oxidation (anammox) (Jetten et al. 1998; Kuenen 2008; Mulder et al. 1995) provide an alternative approach for the complete conversion of ammonia to dinitrogen gas through the cooperation between aerobic and anaerobic ammonium oxidizing bacteria. Due to their reduced oxygen requirements compared to conventional nitrogen removal systems, an increasing number of partial nitritation-anammox systems have been implemented in full-scale WWTPs treating ammonium-rich and municipal wastewaters (Lackner et al. 2014; Liu et al. 2017). Additionally, recently discovered nitrogen-cycling microorganisms, such as ammonia-oxidizing archaea (AOA) (Konneke et al. 2005) and complete ammonia oxidizer (comammox) (Daims et al. 2015; van Kessel et al. 2015), hold potentials for application in biological nitrogen removal WWTPs (Lawson and Lucker 2018; Wang et al. 2021). It should be noted that the key players involved in the biological nitrogen removal process, namely nitrifiers and anammox, are autotrophic microorganisms that can be easily inhibited by relatively high concentration of organic carbon in the wastewater. Therefore, the removal of organic carbon should be prioritized, particularly in the case of anammox systems.

In addition to autotrophic nitrogen-cycling microorganisms, there has been growing interest in microorganisms capable of simultaneous heterotrophic nitrification and aerobic denitrification (HNAD) under aerobic conditions, as they offer potential for alternative nitrogen removal methods (Chen et al. 2020; Joo et al. 2005a). HNAD nitrogen removal is suggested to proceed through the canonical nitrification (NH_4_^+^  → NH_2_OH → NO_2_^−^ → NO_3_^−^) and denitrification process (NO_3_^−^/NO_2_^−^ → N_2_). The direct ammonia oxidation (Dirammox) to N_2_ via hydroxylamine, which was found in *Alcaligenes ammonioxydans* and was distinct from the HNAD process, has further expanded the microbial nitrogen-cycling network (Wu et al. 2021).

As heterotrophic microorganisms, Dirammox bacteria (1) exhibit a rapid growth rate, enabling quick startup of biological nitrogen removal system, and (2) they can also simultaneously remove carbon and nitrogen pollutants from wastewater. Dirammox pathway has been proved to be mediated by enzymes encoded by the gene cluster *dnfT1RT2ABCD*, as demonstrated by physiological and biochemical experiments (Wu et al. 2021, 2022). Genetic analysis further revealed that this functional gene cluster was phylogenetically conserved in genus *Alcaligenes* (Hou et al. 2022). Several bacterial strains belonging to the genus *Alcaligenes* had been isolated from environmental samples, and their nitrogen performance had been characterized using synthetic (Chen et al. 2021; Joo et al. 2005b; Zhang et al. 2022) or real wastewater (e.g., piggery wastewater) (Joo et al. 2006). However, prior to the identification of Dirammox, these isolates may have been wrongly regarded as bacteria involved in the HNAD process. Distribution analysis revealed that approximately 31% *Alcaligenes* strains were derived from WWTPs or wastewater (Hou et al. 2022). The novel nitrogen metabolic pathway and heterotrophic lifestyle of Dirammox bacteria enable them promising candidates for the development of new wastewater treatment systems (Pan and Liu 2023). However, it remains unknown how Dirammox strains be selectively enriched in activated sludge (AS), thereby becoming the dominant microorganisms contributing to nitrogen removal.

In this study, the objective was to get a Dirammox-dominated AS community for biological nitrogen removal. Additionally, we aimed to isolate and characterize new Dirammox strains from the AS. To achieve this, a lab-scale sequencing batch reactor was set up and inoculated with AS obtained from a conventional secondary WWTP. The reactor was operated to treat synthetic wastewater, with the transient selection pressure of hydroxylamine (an intermediate of Dirammox) being utilized to enrich Dirammox strains. To inhibit the activities of anaerobic denitrifiers, the dissolved oxygen (DO) concentration and well suspend biomass in the reactor was controlled and maintained oxic. Changes in microbial community structures were examined using high-throughput 16S rRNA gene sequencing (V3–V4 region). Isolation of bacteria and assessment of nitrogen-removal rates were conducted to identify the potential Dirammox strains. Furthermore, the validation of microorganisms possessing the Dirammox pathway was performed by integrating whole genome sequencing, genomic analysis, and experimental results. Our findings illustrated that Dirammox bacteria could be rapidly selected as the predominant members in AS and may play a crucial role in the nitrogen removal from wastewater. Three Dirammox strains, including one representing a novel species of *Alcaligenes*, were isolated from the AS. The relative abundance, ammonia removal performance, and genomic comparison of these Dirammox strains were investigated. This study highlights the potential for achieving rapid enrichment of Dirammox bacteria in activated sludge systems, providing a foundation for the future engineering applications of Dirammox bacteria in biological nitrogen removal processes.

## Materials and methods

### Reactor operation

A lab-scale reactor with a working volume of 1.6 L was inoculated with AS from the secondary settling tank of wastewater treatment plant at Shandong University (Qingdao, China). The reactor was operated in sequencing batch mode with three reaction cycles per day (8 h per cycle). A fix exchange ratio of 35% was employed to achieve a hydraulic retention time (HRT) of 22.8 h. The 8-h cycle included a 10-min feed phase, a 450-min reaction phase (with aeration turned on after 1 h to slow down hydroxylamine degradation under aerobic condition), a 5-min settling phase, a 5-min discharge phase, and a 10-min idle phase. The reactor was maintained at a temperature of 30℃, while the pH was controlled within the range of 8.29 to 9.09. The speed of stirrer was set to 450 rpm to ensure a homogeneous distribution of substrate and biomass. Heterotrophic nitrification medium (HNM) that used in the previous study (Wu et al. 2021) was modified to increase the microbial diversity in the activated sludge by providing different types of carbon sources. The carbon source was adjusted to a mixture of small molecular acids, and the COD was maintained at 1.9 g/L. Specifically, the carbon source is comprised of the following components (g/L): sodium succinate (0.57), sodium citrate (0.90), sodium malate (0.69), sodium acetate (0.29), and glucose (0.63) (Table S1). To enrich the Dirammox microorganisms from the sludge, hydroxylamine was used as a selective pressure and added simultaneously with the influent water (with an initial hydroxylamine-N concentration of 28 mg/L in reactor). Additionally, to inhibit the activity of anaerobic denitrifiers, DO in the reactor was maintained at relatively high levels, ranging from 7.19 to 7.87 mg/L.

### Analytical methods

Bacterial growth was monitored by measuring OD_600_ (spectrophotometer model BioTek, SynergyH4). Concentrations of ammonium-nitrogen (NH_4_^+^-N), nitrite-nitrogen (NO_2_^−^-N), and nitrate-nitrogen (NO_3_^−^-N) were measured using standard methods recommended by the manufacturer on an Aquakem 600 Analyser (Thermo Scientific). The hydroxylamine content was determined using the 8-quinolinol method, which involved the formation of stable 5,8-quinolinequinone-5-(8-hydroxy-5-quinolylimide) (Frear and Burrell 1955). The TN was determined using Hach kits and the TNT persulfate digestion method.

### Bacterial isolation and phylogenetic analysis

To isolate Dirammox bacteria, AS from the reactor was obtained after 50 and 60 days of operation. Sludge samples (10 ml) were homogenized by beads and mixed with 90 ml of HNM medium. Hydroxylamine was added to the HNM medium to act as selective pressure with a final concentration of 14 mg/L. The mixture was grown on a rotary shaker at 180 rpm and 30℃ for 2 days. The concentration of NH_4_^+^-N was monitored every 12 h, and the enrichment medium was replenished whenever the NH_4_^+^-N was depleted. After 4 days of pre-enrichment, the culture was diluted to 10^−3^ and 10^−5^, plated on a solid separation medium, and incubated at 30℃ for 3 days. After colony growth, colonies exhibiting different sizes and colors were selected and streaked repeatedly on fresh agar plates until pure bacterial were obtained. These colonies were incubated in 48-well plates with addition of HNM medium at 30℃ for 48 h. The strains for further investigation were selected through screening for ammonia oxidation intermediates (i.e., hydroxylamine).

Taxonomic information of the hydroxylamine producing strains was determined based on similarity search of 16S rRNA gene sequences (primer set, 27F-1492R). The resulting 16S rRNA gene was searched against NCBI database using the BLAST (v2.13.0) (Camacho et al. 2009). A phylogenetic tree was then constructed using MEGA 11 (Tamura et al. 2021) to establish the relationships between the screened bacterial strains and other known species from neighboring taxa.

### Ammonia removal experiments

Initially, three bacterial strains were screened for their potential to remove ammonia as Dirammox bacteria. Biodegradation experiments of these three bacteria were then conducted using 250-ml Erlenmeyer flasks with 100 ml working volume of modified HNM (mHNM) to evaluate the growth and ammonia conversion. Overnight precultures of the strains were prepared in LB broth medium. The cells were harvested and resuspended to an optical density of OD_600_ = 1.0, and 1 ml of bacterial solution was transferred into the mHNM medium. The flasks were maintained at 30℃ under constant agitation at 180 rpm in a temperature-controlled orbital incubator shaker. Cultures were sampled at appropriate time points to measure cell growth (OD_600_), and the concentrations of total nitrogen (TN), hydroxylamine-N, NH_4_^+^-N, NO_2_^−^-N, and NO_3_^−^-N. The composition of mHNM included (g/L): (NH_4_)_2_SO_4_ (0.66), C_4_H_4_Na_2_O_4_·6H_2_O (4.72), KH_2_PO_4_ (1.5), Na_2_HPO_4_·12H_2_O (7.9), MgSO_4_·7H_2_O (0.2) (Table S1). Trace element solution was prepared according to Joo et al. (2005c) and added to the mHNM medium (2 ml/L). The initial pH of the medium was adjusted to 7.5–8 using NaOH/HCl (Table S1). All experiments were performed in triplicate, and all values shown in the figures represent the means ± standard error of mean (SEM) for the triplicates.

To determine the gaseous nitrogen products of these three bacterial strains, a 1-ml aliquot of the precultured cells (OD_600_ = 1.0) in LB medium was harvested and transferred into 100-ml sealed bottles containing 10 ml of HNM containing 5 mM (^15^NH_4_)_2_SO_4_. The headspace of the bottles was purged with 4:1 He/O_2_ (20% O_2_, aerobic conditions) using a gas displacement system. After 48 h of incubation, cultures were sampled to determine cell growth (OD_600_) and the concentrations of hydroxylamine, TN, NH_4_^+^-N, NO_2_^ˉ^-N, and NO_3_^ˉ^-N. At the same time, the production of ^15^N_2_ and ^15^N_2_O was measured using GC/MS (7890A/5975C, Agilent) equipped with a CP-Molsieve 5A Plot column (25 m × 0.32 mm × 30 μm, Agilent, USA). All experiments were performed in triplicate, and the values shown in the figures represent the means ± standard error of mean (SEM) for the triplicates.

### Microbial population dynamics

Sampling was conducted over 2-month from September to November 2023, with the aim to investigate changes in the microbial community structure within the reactor under the selective pressure of hydroxylamine. Total genomic DNA was extracted from the collected samples using the CTAB/SDS method. The V3–V4 region of the 16S rRNA gene was amplified by PCR using the primers 341F (CCTACGGGRSGCAGCAG) and 806R (GGACTACHVGGGTWTCTAAT) (Takahashi et al. 2014), and the resulting amplicons were sequenced by the Illumina NovaSeq platform provided by a commercial service provider (Novogene, Tianjin, China).

The amplicon sequences were trimmed using fastp (v0.23.2) (Chen et al. 2018) with default parameters. The paired sequences were demultiplexed and analyzed via QIIME2 (v2021.4.0) (Bolyen et al. 2019). The DADA2 plugin (v2021.4.0) (Callahan et al. 2016) was used to create a feature table with representative sequences (features) and their corresponding frequencies. Taxonomic assignment was performed using the QIIME2 “feature-classifier” plugin’s “classify-sklearn,” after training the classifier on the Greengenes2 database (v.2022.10) (McDonald et al. 2023) with 341F/806R primers.

To figure out the representative feature of the isolated bacterial strains, the representative sequences generated by QIIME2 were exported and used to build a curated database by BLAST. We, then, compared the full length 16S rRNA gene sequences of the isolated bacterial strains with the amplified sequences of 16S rRNA V3–V4 region using blastn (v2.13.0). Feature sequences that were identical to the 16S rRNA gene of isolated strains were selected as representative features of bacterial isolates.

### Genome sequencing and genome recovery

The total DNA of Dirammox strains was divided into two equal parts, with one part was used to shotgun sequencing on Illumina Novaseq platform by a commercial service provider (Novogene, Tianjin, China) to generate 150 bp paired-end reads with a 350-bp insert size. The other part was sequenced using a Nanopore GridION sequencer (Oxford Nanopore Technology, ONT, UK). The total DNA of other isolates without nitrogen removal potential was only sequenced using GridION sequencer to obtain reference-quality draft genome. Illumina short reads with an average base-quality score ≤ 30 were filtered using fastp, resulting in an average of 3327.6 Mbp data for each isolate. For the ONT long reads, only reads longer than 1 Kbp and average-based quality score ≥ 10 were retained, resulting in an average of 135 Mbp data for each isolate. The quality controlled short and long reads of Dirammox strains were imported to NanoPhase (v0.2.3) to perform hybrid assembly with default parameters. The draft genomes of other isolates were assembled using “–long_reads_only” mode of NanoPhase (v0.2.3) (Liu et al. 2021, 2022). The qualities and taxonomic affiliations of obtained genomes were estimated using CheckM (Parks et al. 2015) and GTDB-Tk (v0.2.1.1, database release 89) (Chaumeil et al. 2019; Parks et al. 2018), respectively. The phylogenetic tree was constructed using GTDB-Tk for the genomes of three isolated *Alcaligenes* strains and 59 high-quality *Alcaligenes* genomes collected from NCBI. *Paenalcaligenes* was selected as outgroup. The Digital DNA:DNA hybridization (DDH) and average nucleotide identity (ANI) between newly recovered Dirammox genomes and three previously reported Dirammox genomes were determined using Genome-to-Genome Distance Calculator (v3.0) (Meier-Kolthoff et al. 2022) and FastANI (v1.33) (Jain et al. 2018) with default parameters, respectively.

### Genome annotation and comparative genomic analysis

The open reading frames (ORFs) for the isolates’ genomes were predicted using Prodigal (v2.6.3) (Hyatt et al. 2010) and subsequently annotated using Prokka (v1.14.6) (Seemann 2014). The completeness of metabolic pathways for Dirammox was estimated using EnrichM (v0.5.0; https://github.com/geronimp/enrichM). High-quality reference genomes of *Alcaligenes* were collected from NCBI and combined with the newly recovered genomes in the present study for pangenome analysis. To identify the *dnf* gene clusters, the predicted ORFs of the newly recovered genomes of *Alcaligenes* were searched against the *dnf* databases of reported Dirammox genomes using blastn.

## Results

### Nitrogen removal performance of the lab-scale reactor

**Fig. 1:**
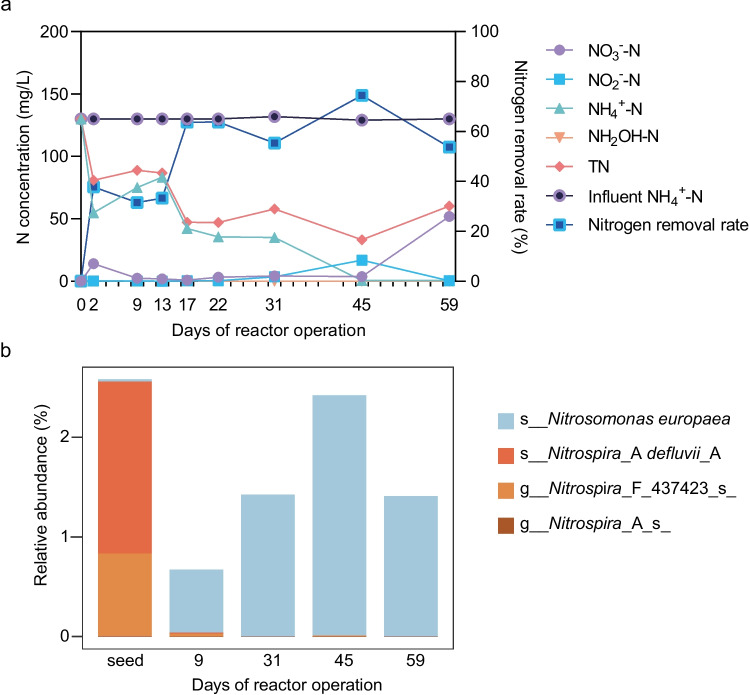
Nitrogen removal performance of the lab-scale reactor (**a**) and dynamics of microorganisms involved in nitrification (**b**)

To inhibit the activity of anaerobic denitrification, the DO in the lab-scale reactor was maintained at a high level, ranging from 7.19 to 7.87 mg/L (Table S2). As shown in Fig. 1a, the ammonia in the synthetic wastewater was completely oxidized after approximately 40 days of reactor startup. During the early phase of reactor startup (from day 0 to day 30), the average concentrations of NO_2_^−^-N and NO_3_^−^-N in effluent were 0.26 and 1.69 mg/L, respectively. Starting from the 40th day, NO_3_^−^-N in the effluent gradually increased to 51.90 mg/L and stabilized thereafter (Table S2). The sequencing batch operational mode adopted in this study retained more than half of the treated water after each cycle, which could account for the increase in NO_3_^−^-N. In contrast, NO_2_^−^-N in the effluent exhibited low concentration, with an average concentration of 8.67 mg/L. The overall nitrogen removal rate in this reactor achieved a relative stable state after 1 month of operation, with an average nitrogen removal rate (TN based) of 64.1%.

### Enriching ammonia-oxidizing bacteria with addition of hydroxylamine

The synthetic HNM influent had a discernible impact on the overall AS community structure, as evidenced by the observed changes in microbial community composition over time (Fig. S1). The two components of principal coordinate analysis (PCoA) with species abundance explained 91.45% variance. Community composition in the seed sludge shifted to a distinct state after only 9 days of operation. Interestingly, the microbial communities of AS collected from day 31, day 45, and day 59 shared high similarity and formed a cluster, suggesting the microbial community state was stable after one month of operation.

In terms of bacterial taxa, the phylum *Proteobacteria* was found to be the most abundant bacteria across all analyzed AS samples. The phyla *Bacteroidota*, *Actinobacteriota*, *Chloroflexota*, and *Acidobacteriota* exhibited high abundance in the seed sludge, while their abundances dramatically decreased in reactor treating HNM with addition of hydroxylamine. In contrast, the relative abundances of *Proteobacteria* and *Deinococcota* were enriched in the reactor (Fig. S2a). Despite *Proteobacteria* being the predominate phylum in both seed sludge and reactor AS, the dominant genera within *Proteobacteria* differed substantially between the two. Only one of the top 10 abundant genera from *Proteobacteria* in seed sludge (relative abundance ≥ 1%) was found to be abundant in reactor (≥ 1%), while the others became rare populations (< 0.01%) after treatment with synthetic wastewater (Table S3). The bacteria from the genera of *Thauera*, *Alcaligenes*, *Comamonas*, *Aquabacter*, and JAABTL01 (belonging to the family *Trueperaceae*), which were rare members in seed sludge, were identified as abundant populations (Table S3 and Fig. S2b).

Regarding the autotrophic nitrifiers in the reactor (Fig. 1b), nitrite-oxidizing bacteria (NOB) (2.56%) predominated over ammonia-oxidizing bacteria (AOB) (0.02%) in the seed sludge. The addition of hydroxylamine at the beginning of each reaction cycle increased the abundance of AOB and efficiently repressed NOB in the reactor. The average abundance of AOB and NOB in the reactor was 1.47% and 0.012%, respectively, suggesting that the oxidation of nitrite to nitrate via NOB may be efficiently inhibited.

### Hydroxylamine producing bacteria screening and *dnf* gene cluster identification

**Fig. 2:**
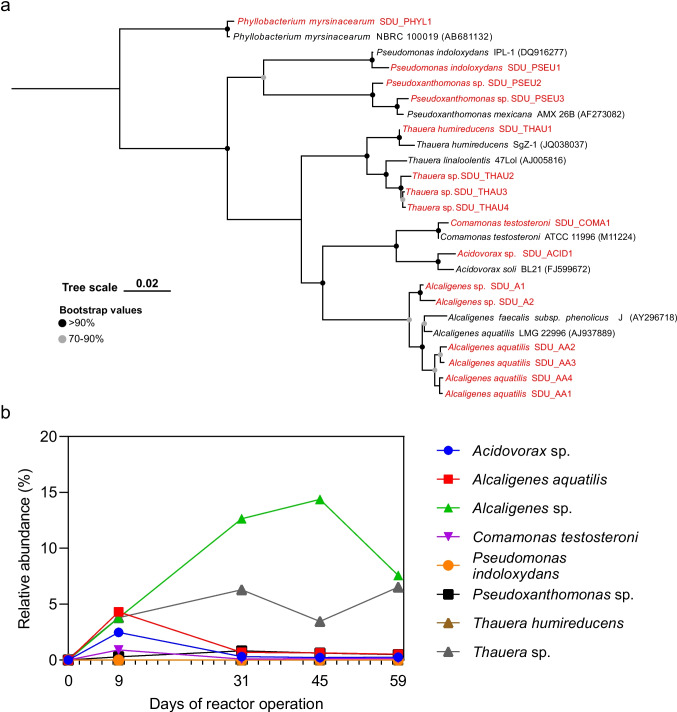
Isolated bacterial strains and their relative abundance across the studied activated sludge. **a** Phylogenetic tree inferred based on the full-length 16S rRNA genes of these newly isolated bacterial strains and reference sequences. **b** The relative abundance of features (amplicon sequences) representing the newly isolated bacterial strains across the studied activated sludge samples. The relationships between features and bacterial isolates were determined based on sequence similarity. The feature sequences that are identical to the 16S rRNA gene of isolates are selected as representative taxa

After achieving stable nitrogen removal performance and community state, we conducted bacterial isolation to harness Dirammox bacteria in the reactor. Two AS samples collected on the 50th and 60th days were used for bacterial isolation. A total of 200 colonies were picked from HNM agar plates based on their sizes and colors. These colonies were incubated in 48-well plates with addition of HNM medium at 30℃ for 48 h. The ammonia oxidation intermediates (i.e., hydroxylamine) in wells were screened, resulting in 16 bacterial strains with detectable hydroxylamine. These isolates were affiliated with eight different species with cutoff of 98.7% 16S rRNA gene identity (including four novel species), namely, *Acidovorax* sp*.*, *Comamonas testosteroni*, *Pseudomonas indoloxydans*, *Pseudoxanthomonas* sp., *Thauera humireducens*, *Thauera* sp*.*, *Alcaligenes aquatilis*, and *Alcaligenes* sp*.*, belonging to the class *Gammaproteobacteria* (Fig. 2a).

We examined the relative abundances of these isolates by comparing the full-length 16S rRNA genes of isolates to the feature sequences. As shown in Fig. 2b, the representative feature of the isolates accounted for an average of 17.83% of the whole microbial communities in reactor. Within AS taken from day 9 to day 59, isolates from *Alcaligenes aquatilis*, *Thauera* sp*.*, and *Alcaligenes* sp*.* were identified as abundant community members in the reactor. Notably, the three *Alcaligenes* isolates comprised approximately 13.34% of the AS microbial community after 1 month of operation. *A. aquatilis* and *A.* sp*.* were simultaneously enriched after only nine days of operation, reaching relative abundances of 4.32% and 3.77%, respectively. Interestingly, *A.* sp*.* isolate outnumbered *A. aquatilis* and emerged as the dominant *Alcaligenes* species, exhibiting an average relative abundance of 11.53% from day 31 to day 59. Other isolated bacterial strains were observed as rare populations in the analyzed AS, except for isolates from *Comamonas testosteroni* and *Acidovorax* sp*.*, which were once identified as abundant organisms in the AS collected on day 9. It is noteworthy that all these isolates were classified as rare populations in the seed AS sample, with a total abundance of 0.14%, which was consistent with the observed shift of microbial community compositions in the reactor.

To figure out whether these isolates are putative Dirammox strains, we sequenced and assembled nine isolated bacteria, including three *Alcaligenes* isolates and six representative isolates of other six species (Table S4). The complete Dirammox *dnf* gene cluster was identified in only three isolates from the genus *Alcaligenes* (*A. aquatilis* SDU_AA1, *A. aquatilis* SDU_AA2, and *A.* sp*.* SDU_A2). While the complete *dnf* gene cluster could not be identified in the other isolates, *dnfBCD* and/or *dnfTR* could be completely or partial identified in the other isolates (Fig. S3). However, their ammonia metabolic potential required experimental validation. As for the genes for denitrification process, the *Alcaligenes* strains that dominant in AS samples encoded genes necessary for denitrification from nitrite to dinitrogen gas. Besides, the less abundant bacteria from *Thauera* sp*.* SDU_THAU2 and *Acidovorax* sp. SDU_ACID1 also encoded genes for complete denitrification (Fig. S3).

### Capability of ammonia removal

**Fig. 3:**
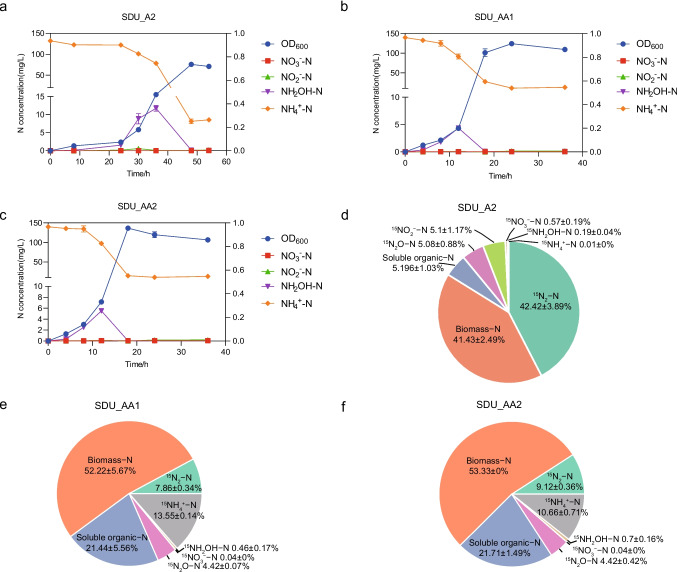
Ammonia removal characteristics of the newly isolated Dirammox strains. Growth and ammonia conversion patterns of SDU_A2 (**a**), SDU_AA1 (**b**), and SDU_AA2 (**c**) in mHNM. Nitrogen balance results of SDU_A2 (**d**), SDU_AA1 (**e**), and SDU_AA2 (**f**). The nitrogen balance experiments were conducted in HNM containing 5 mM (^15^NH_4_)_2_SO_4_ with initially 4:1 of He/O_2_ (20% of O_2_) in the headspaces

Based on the genetic analysis, we conducted further experiments to investigate the ammonia removal ability of the isolates with *dnf* gene cluster (i.e., SDU_AA1, SDU_AA2, and SDU_A2). As shown in Fig. 3a, b, and c, the growth curves and nitrogen metabolism of these three bacteria were different from each other. Compared to the two *A. aquatilis* isolates (~ 12 h), SDU_A2 displayed the longest lag phase (24 h) in the mHNM medium. Although SDU_AA1 and SDU_AA2 had similar lag phases, SDU_AA2 displayed a higher growth rate (0.106 h^−1^) than SDU_AA1 (0.104 h^−1^) between 12 and 18 h. The OD_600_ of SDU_AA1 reached its highest value (0.915) after 24 h, while SDU_AA2 reached its peak (0.956) after 18 h. Due to the extended lag phase of SDU_A2, its highest OD_600_ was identified after 48 h.

During the lag phase, the concentration of NH_4_^+^-N remained relatively stable for all three *Alcaligenes* strains. NH_4_^+^-N levels rapidly decreased upon entering the log phase and reached a stable state during the decline phase. Despite SDU_A2 having the longest lag phase and the lowest cell density, the final concentration of NH_4_^+^-N was much lower in the SDU_A2 culture (8.55 mg/L) compared to SDU_AA1 (12.67 mg/L) and SDU_AA2 (13.0 mg/L). With the decrease in NH_4_^+^-N, we observed accumulations of hydroxylamine, although the maximum concentrations differed among the isolates. SDU_A2 exhibited the highest accumulation of hydroxylamine at 11.75 mg/L, which was twofold higher than that in SDU_AA1 and SDU_AA2 cultures. Notably, the average concentrations of NO_3_^−^-N and NO_2_^−^-N were below 0.3 mg/L throughout the entire reaction period for these three *Alcaligenes*. Furthermore, we measured the concentrations of total nitrogen at the end point of the reactions. ^15^N-labled nitrogen balance experiments of these three *Alcaligenes* strains further confirmed that SDU_A2 exhibited the highest nitrogen removal rate (*P* < 0.05). As shown in Fig. 3d, SDU_A2 converted 42.42% and 5.08% of ^15^N-labeled ammonium to ^15^N_2_ and ^15^N_2_O, respectively. In contrast, SDU_AA1 and SDU_AA2 only converted 7.86% and 9.12% of ^15^N-labeled ammonium to ^15^N_2_, respectively. The ratios of biomass and soluble organic nitrogen products in SDU_AA1 and SDU_AA2 were significant higher (*P* < 0.05) than those of SDU_A2 (Fig. 3e and f).

### Comparative genomics analysis

Complete bacterial genomes (circular chromosome) of SDU_AA1, SDU_AA2, and SDU_A2 were obtained using hybrid assembly of Illumina short reads and Nanopore long reads (Table S5). Complete genomes of three previously reported Dirammox strains (*A. faecalis* subsp. *phenolicus* DSM16503, *A. ammonioxydans* HO-1, and *A. faecalis* strain JQ135) were included in the comparative genomics analysis. The total genome sizes of these six Dirammox varied from 3.62 to 4.29 Mbp (Table S5). In comparison to the average genome size (4.0 ± 0.19 Mbp (average ± standard deviation)) and encoded genes (3644 ± 192 (average ± standard deviation)) of the other five Dirammox strains, SDU_A2 exhibited a smaller genome size (3.62 Mbp) and fewer encoded genes (3182) (Table S4). SDU_AA1 and SDU_AA2 are almost identical with an average nucleotide identity (ANI) of 99.99% and digital DNA:DNA hybridization (DDH) of 100% (Table S6), respectively.

**Fig. 4:**
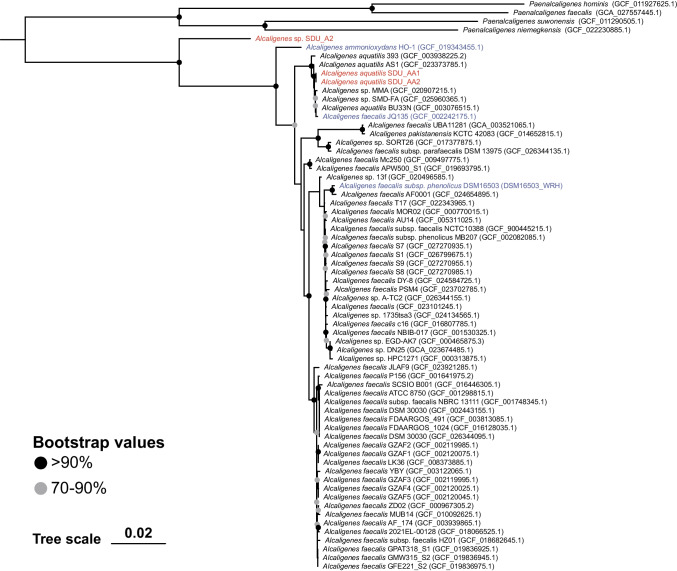
Phylogenomic tree of *Alcaligenes*. A total of 62 high-quality genomes of Alcaligenes were collected from NCBI, the reported dirammox that characterized in the present study and the newly isolated *Alcaligenes* are colored in blue and red, respectively. *Paenalcaligenes* was selected as outgroup. The phylogenomic tree was inferred using GTDB-Tk (v0.2.1.1)

As shown in the phylogenomic tree constructed by 62 high-quality genomes of *Alcaligenes* (Fig. 4), SDU_AA1 and SDU_AA2 showed close phylogenetic relationships with reported strains of *A. aquatilis*. The most abundant *Alcaligenes* isolated from the AS (i.e., SDU_A2) is basal to *Alcaligenes*. The recovered 16S rRNA gene of SDU_A2 was searched against the NCBI 16S rRNA gene database. The best hit for SDU_A2 16S rRNA gene was a sequence from *A. faecalis* strain NBRC 13111 sharing an identity of 98.3%. The ANI and DDH between SDU_A2 and the phylogenetically closed *Alcaligenes* strains (i.e., HO-1, Fig. 4) were 80.32% and 26.1% (Table S6), respectively. According to the phylogenetic and genomic distance analyses, SDU_A2 may represent a strain of a new *Alcaligenes* species.

**Fig. 5:**
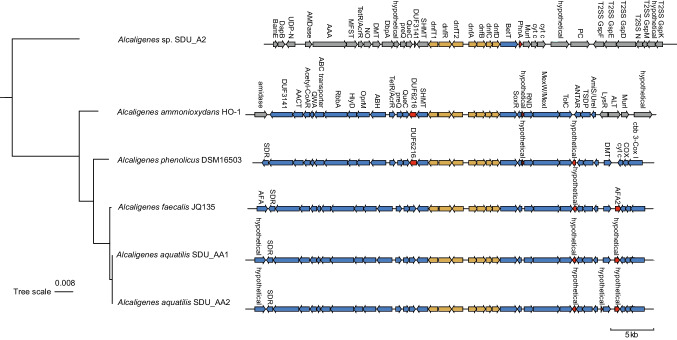
The gene arrangement of the *dnf* gene cluster and its neighboring genes in six Dirammox strains (three isolated in the present study and three previously reported). The phylogenomic tree depicting the evolutionary relationships is shown on the left side. In the gene arrangement map, blue represents the homologous of genes flanking the *dnf* gene cluster (yellow colored), red indicates the occurrence of insertion events, and gray indicates a different gene arrangement compared to the other strain

Canonical genes coding for enzymes involved in ammonia oxidation and nitrite oxidation were not identified in any of the studied *Alcaligenes* genomes. In contrast, the recently reported Dirammox gene cluster *dnfT1RT2ABCD* was found in these genomes (Fig. 5). It is notable that the flanking regions of *dnf* gene cluster were highly conserved between the *Alcaligenes* strains except for SDU_A2. Although, HO-1 and DSM16503 are affiliated with *A. ammonioxydans* and *A. faecalis*, respectively, these two bacteria encoded highly similar gene content with bacteria from *A. aquatilis* (i.e., JQ135, SDU_AA1, and SDU_AA2). Moreover, the gene arrangement and positions adjacent to *dnf* gene cluster in DSM16503, JQ135, SDU_AA1, and SDU_AA2 are nearly identical, except for two insertions identified in DSM16503 and SDU_AA1 (Fig. 5). Two insertion events (DUF6216 family and hypothetical proteins) were found when comparing DSM16503 and three strains (JQ135, SDU_AA1, and SDU_AA2) from *A. aquatilis*. However, the functions of these two inserted genes remain unknown. One hypothetical protein was identified in DSM16503, JQ135, SDU_AA1, and SDU_AA2, positioned between genes encoding the outer membrane protein TolC and REC and RNA-binding antiterminator (ANTAR), which was not identified in HO-1. Additionally, one hypothetical protein was only found in SDU_AA1, positioned between genes encoding the urea channel and putative amide transporters (UreI/AmiS) and permease of the drug/metabolite transporter (DMT). Apart from the unknown or hypothetical proteins located near the *dnf* gene cluster, the genes in the conserved gene cluster of HO-1, DSM16503, JQ135, SDU_AA1, and SDU_AA2 primarily coded for membrane proteins involved substrate transportation (e.g., choline (BetT), multidrug (MexW/MexI), urea/amide (UreI/AmiS)), amino acid metabolism (e.g., glutamine aminotransferase, serine hydroxymethyltransferase, and PLP-dependent aminotransferase), and electron transport chain (e.g., cytochrome c oxidases). However, the gene flanking regions of the *dnf* gene cluster in SDU_A2 were distinct from that in other five Dirammox strains, containing several genes involved in type II secretion systems.

Comparative genomic analysis revealed the differences in metabolic potentials among these six studied Dirammox strains. As expected, SDU_A2 possessed fewer complete metabolic modules (85) compared to the other strains (averaging 99) (Table S7). The missing metabolic modules in SDU_A2 were associated with membrane transportation (e.g., phosphonate, capsular polysaccharide, heme, and multidrug), amino acid biosynthesis (valine/isoleucine), and vitamin metabolism (e.g., nicotinate degradation). The degradation potentials for aromatic compounds (e.g., benzene and benzoate) were found in these Dirammox strains except SDU_A2. Additionally, a two-component system (i.e., QseC-QseB), involved in the regulation of multiple bacterial behaviors by regulating quorum sensing, was only absent in SDU_A2. Despite the absence of certain KEGG modules were absent in SDU_A2, it showed genetic potentials for tetrahydrofolate biosynthesis from GTP and multidrug resistance via the AdeS-AdeR two-component regulatory system. Regarding the reported Dirammox strains, we found five metabolic modules exclusively present in HO-1. These conserved modules related to antibiotic resistance (e.g., streptomycin, tetracycline), cysteine biosynthesis (from serine to cysteine), and GABA (gamma-aminobutyrate) shunt. Additionally, all genes necessary for GABA biosynthesis were encoded by HO-1 and DSM16503.

## Discussion

Hydroxylamine has been confirmed as intermediate compound in the Dirammox pathway, transiently accumulating during ammonia conversion (Wu et al. 2021; Xu et al. 2022). Besides its involvement in the Dirammox process, hydroxylamine is also a highly reactive intermediate compound produced by AOA (Konneke et al. 2005; Stahl and de la Torre 2012), AOB (Caranto and Lancaster 2017) and comammox (Daims et al. 2015; van Kessel et al. 2015). Previous studies have demonstrated that the addition of hydroxylamine can enhance the growth of AOB by accelerating the ammonium uptake rate (Chandran and Smets 2008; Harper et al. 2009). However, the addition of hydroxylamine has been reported to inhibit NOB (Cao et al. 2017; Kindaichi et al. 2004). The addition of hydroxylamine can lead to a decrease in the oxidation–reduction potential (ORP) and then create a reductive environment that has a more pronounced inhibitory effect on NOB than AOB (Sui et al. 2020). Furthermore, the addition of hydroxylamine has been observed to increase the concentration of NO in the liquid phase (Zhao et al. 2021), which could strongly inhibit the growth of *Nitrospira* (Courtens et al. 2015). Consistent with these findings, the dynamics of microbial community in our study revealed the inhibition of NOB and promotion of AOB after treated wastewater with the addition of hydroxylamine at the beginning of each reaction cycle. Despite the increase of AOB following hydroxylamine addition, their relative abundance was below 2.5% in most of the AS samples under the high levels of organic carbon (1.9 g/L COD). Moreover, the high DO levels in the studied reactor could efficiently inhibit the activities of anaerobic denitrification. These results suggested the nitrogen removal of the reactor might be attributed to the presence of novel nitrogen-cycling microorganisms.

The microbial community profiling showed the rapid enrichment of *Alcaligenes* as the dominant population in the lab-scale AS system treating HNM with the addition of hydroxylamine. Considering the widespread distribution of Dirammox pathway among *Alcaligenes* species (Hou et al. 2022), Dirammox bacteria may contribute to the nitrogen removal in the studied reactor. Representative *Alcaligenes* isolates were obtained for ammonia conversion experiments. The experimental results demonstrated that all three *Alcaligenes* strains exhibited nitrogen removal activities under aerobic condition. Moreover, the most abundant one displayed the highest nitrogen removal rate. In summary, our study provided evidence that the addition of hydroxylamine in each reaction cycle, coupled with the maintenance of relatively high DO levels, and effectively enriched Dirammox strains in AS systems. While HNM is commonly used to enrich heterotrophic ammonia oxidizers, our study did not address whether the addition of hydroxylamine to HNM could accelerate the enrichment process of Dirammox, as no control experiment (without hydroxylamine) was conducted in the present study. Additionally, it is important to note that hydroxylamine in real environment is toxic and unstable. Therefore, hydroxylamine may serve as a selective agent for the startup of a Dirammox-dominated activated sludge system.

Genetic analysis of these three novel Dirammox strains in this study confirmed the universal distribution and phylogenetic conservation of the *dnfT1RT2ABCD* gene cluster in *Alcaligenes* (Hou et al. 2022; Wu et al. 2021; Xu et al. 2022). Furthermore, we observed a highly similar gene arrangement surrounding the gene cluster among the five studied Dirammox strains, which belong to four different species (*A. faecalis*, *A.* sp*.*, *A. ammonioxydans*, and *A. aquatilis*). Based on the genetic annotation of the conserved genes, it is suggested that the metabolism of organic nitrogen such as urea and amide (e.g., glutamine) may be import for Dirammox process. However, we speculate that this conserved flanking regions of *dnf* gene cluster may not have a direct correlation with the Dirammox activities since SDU_A2, which showed divergent gene content near the *dnf* gene cluster, also exhibited relatively high nitrogen removal rate.

Regarding metabolic potentials, SDU_A2, as the predominant Dirammox strain and a putative new species of *Alcaligenes*, possessed fewer complete metabolic modules compared to the other studied Dirammox strains. The missing metabolic modules in SDU_A2 were related to amino acid biosynthesis and vitamin metabolism, which may explain its longer lag period under pure cluster experiments. As discussed in previous studies, bacteria with reduced genomes may have selective advantages in stable environments (Brewer et al. 2016; D'Souza et al. 2014; Johnson et al. 2020), suggesting that SDU_A2 may acquire essential metabolites from other associated microorganisms in the AS systems and achieve higher fitness compared to the other Dirammox strains (SDU_AA1 and SDU_AA2). Although Dirammox strains can be selectively enriched as predominant populations in the lab-scale reactor, the observed fluctuation in the abundance *Alcaligenes* suggested a lack of stability in the community state. Therefore, further investigations are required to optimize operational parameters for the maintenance of community stability and elucidate the underlying mechanisms that enable Dirammox bacteria gain fitness advantages over other microbial members.

In conclusion, the selective enrichment of Dirammox strains in AS is a crucial aspect for its engineered application. Having observed the successful enrichment of Dirammox bacteria by the modified HNM solution with addition of hydroxylamine, this study offers a foundation to further evaluate the feasibility of employing Dirammox for simultaneous nitrogen and carbon removal from wastewater. In the studied reactor, an isolate representing novel Dirammox species of the genus *Alcaligenes* was identified as the most abundant Dirammox strain. Comparative genomic analysis indicated that Dirammox strain with streamlined genome might gain fitness advantage in the complex AS system. The molecular mechanism underlying the distinct activities of Dirammox strains remain to be elucidated.

## Supplementary Information

Below is the link to the electronic supplementary material.

Supplementary file1 (PDF 372 KB)

Supplementary file2 (XLSX 178 KB)

## Data Availability

The original 16S rRNA gene sequence data and genome sequence data in this study were deposited at the NCBI by accession number PRJNA1054365. *Alcaligenes sp.* SDU_A2, *Alcaligenes aquatilis* SDU_AA1, and *Alcaligenes aquatilis* SDU_AA2 are deposited in the China General Microbiological Culture Collection Center (CGMCC) under the accession number CGMCC 1.61902, CGMCC 1.61903, and CGMCC 1.61904, respectively.
