# Supplementary material for: Dirammox-dominated microbial community for biological nitrogen removal from wastewater
Source: Appl Microbiol Biotechnol. 2024 Jun 21;108:389. doi: 10.1007/s00253-024-13214-2 (PMC11192851; doi:10.1007/s00253-024-13214-2)
Supplement: Supplementary file 1 [file 253_2024_13214_MOESM1_ESM.pdf]

Applied Microbiology and Biotechnology

Dirammox-dominated microbial community for biological nitrogen removal from wastewater

Yu Hu[1], Yulin Wang[*1], Runhua Wang[2,3], Xiaokang Wang[2,3], Shuang-Jiang Liu[*1,2,3]

[1]. State Key Laboratory of Microbial Technology, Shandong University, Qingdao, P. R. China.

[2]. State Key Laboratory of Microbial Resources, Institute of Microbiology, Chinese Academy of Sciences, Beijing, P. R. China.

[3]. University of Chinese Academy of Sciences, Beijing, P. R. China.

[*]Corresponding authors. E-mail address: wangyulin@sdu.edu.cn (Yulin Wang); liusj@sdu.edu.cn (Shuang-jiang Liu), Tel.: +86 0532-58632369

The supporting information contains 3 figures and 5 pages in total.

Table legends (in supplementary datasets)

Table S1. Heterotrophic nitrification medium (HNM) and trace element solution recipes.

Table S2. Summary of operational parameters and sludge characteristics.

Table S3. Summary of microbial community compositions (genus level) in seed and reactor sludge.

Table S4. Genomic summary of the bacteria isolated from the activated sludge treating synthetic HNM influent.

Table S5. Characteristics of the six studied Dirammox bacteria.

Table S6. **A**verage nucleotide identity (ANI) and Digital DNA:DNA hybridization (DDH) comparison among the six studied Dirammox strains.

Table S7. Summary of KEGG module completeness in the six studied Dirammox bacteria.

Figure S1. Principal coordinate analysis (PCoA) of the studied activated sludge samples based on Bray-Curtis distances of community structure at species level.

Figure S2. Principal coordinate analysis (PCoA) of the studied activated sludge samples based on Bray-Curtis distances of community structure at species level.

Figure S3. Presence of Dirammox *dnf* gene cluster and denitrification genes across the isolated bacterial strains.

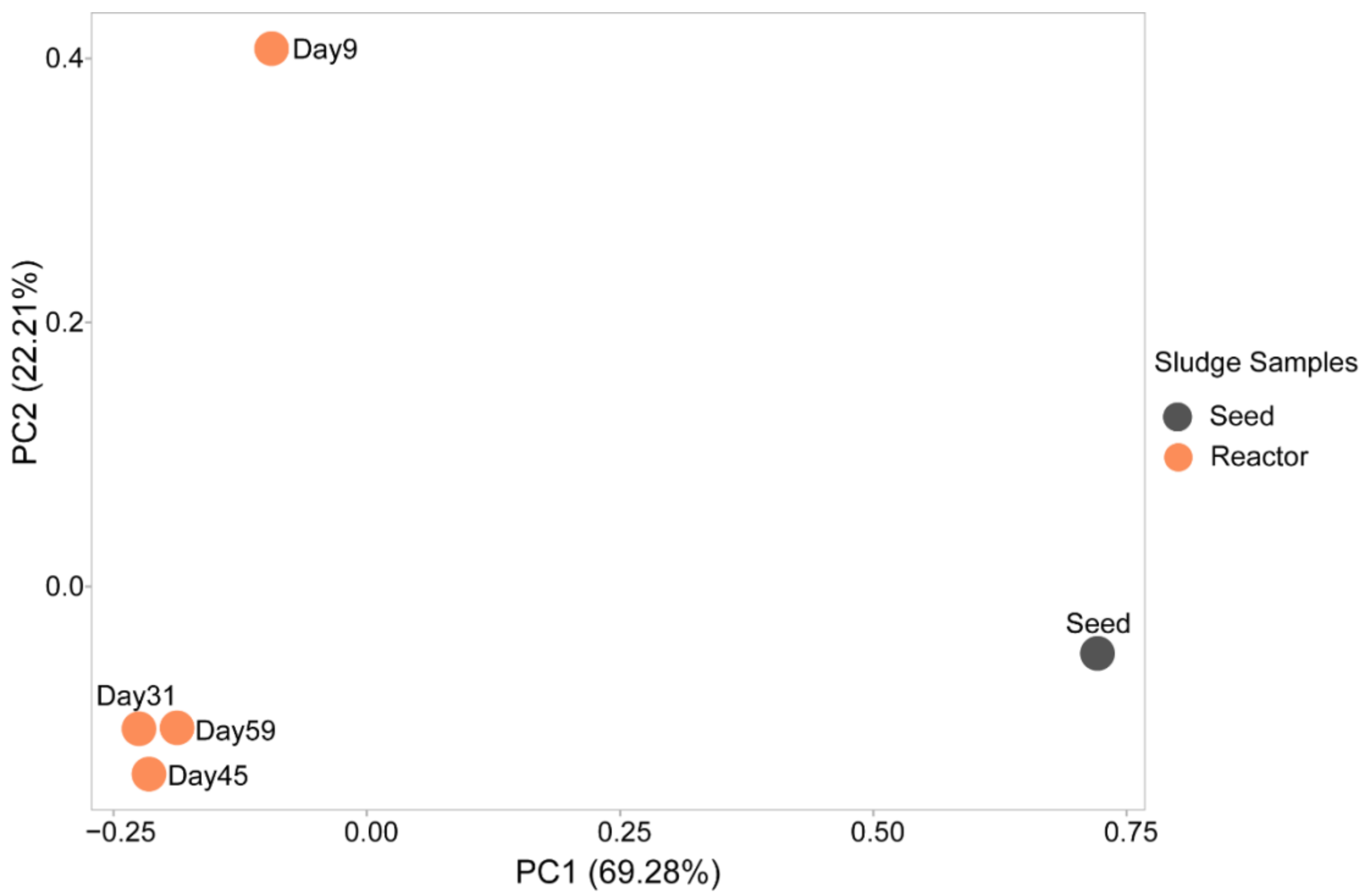

Figure S1. Principal coordinate analysis (PCoA) of the studied activated sludge samples based on Bray-Curtis distances of community structure at species level.

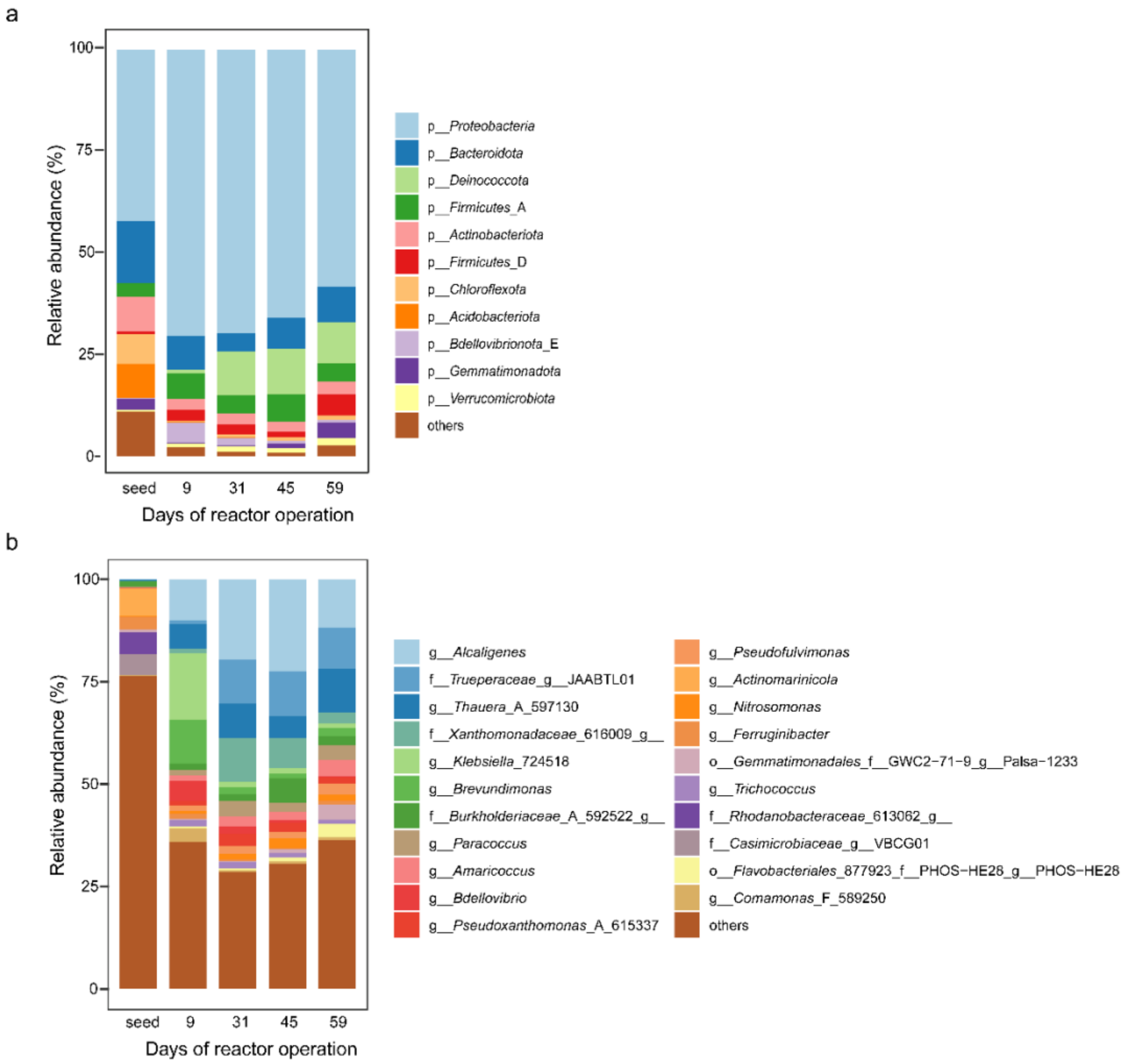

Figure S2. Microbial community compositions at phylum (a) and genus (b) levels. Family names of the genera that cannot be assigned to specific lineages are clarified.

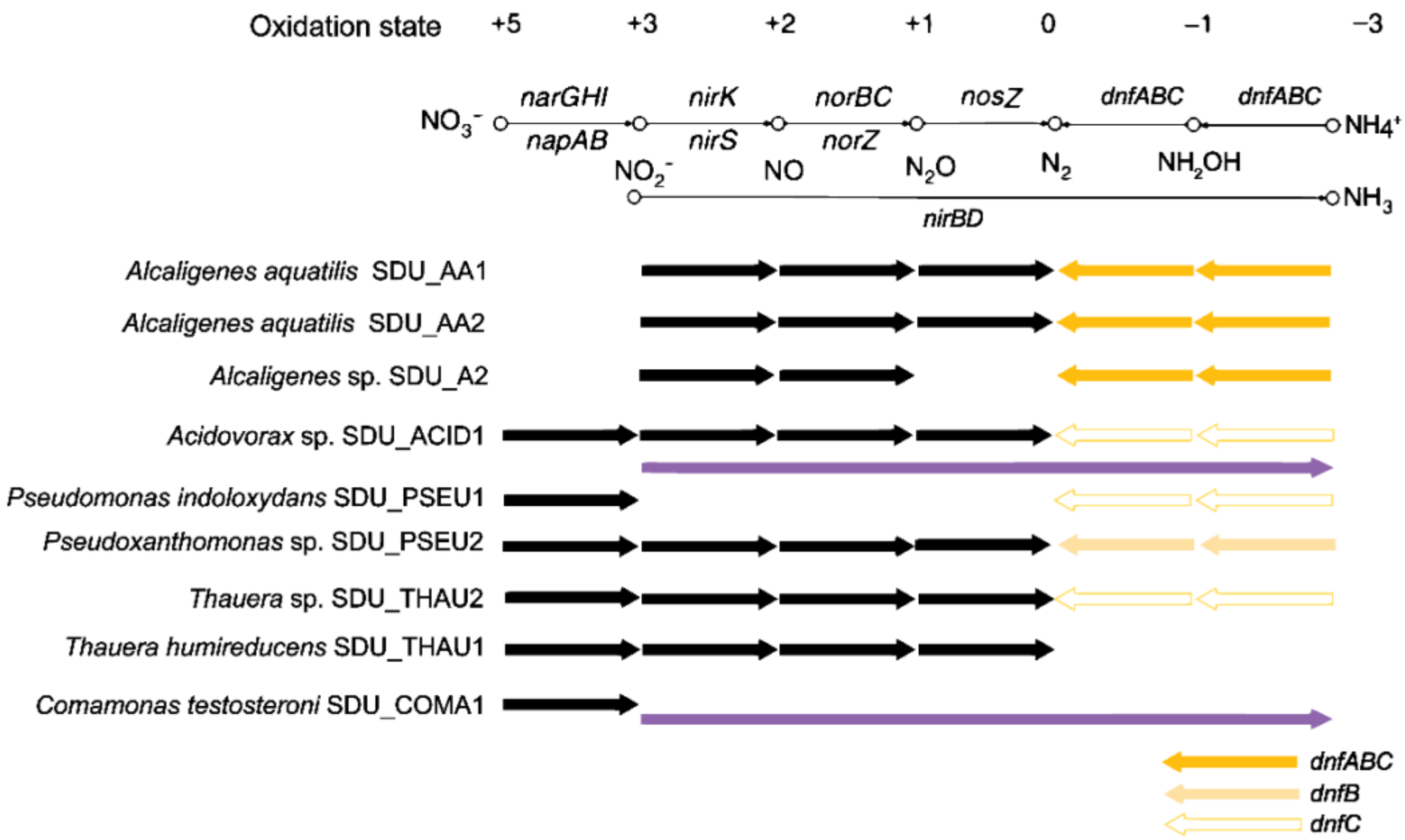

Figure S3. Presence of Dirammox *dnf* gene cluster and denitrification genes across the isolated bacterial strains.
